# Cardiac Myosin Binding Protein-C Phosphorylation Modulates Myofilament Length-Dependent Activation

**DOI:** 10.3389/fphys.2016.00038

**Published:** 2016-02-15

**Authors:** Ranganath Mamidi, Kenneth S. Gresham, Sujeet Verma, Julian E. Stelzer

**Affiliations:** ^1^Department of Physiology and Biophysics, School of Medicine, Case Western Reserve UniversityCleveland, OH, USA; ^2^Department of Horticultural Science, Institute of Food and Agricultural Sciences Gulf Coast Research and Education Center, University of FloridaWimauma, FL, USA

**Keywords:** cardiac myosin binding protein-C, phosphorylation, protein kinase A, stretch-activation, cross-bridge kinetics, skinned myocardium

## Abstract

Cardiac myosin binding protein-C (cMyBP-C) phosphorylation is an important regulator of contractile function, however, its contributions to length-dependent changes in cross-bridge (XB) kinetics is unknown. Therefore, we performed mechanical experiments to quantify contractile function in detergent-skinned ventricular preparations isolated from wild-type (WT) hearts, and hearts expressing non-phosphorylatable cMyBP-C [Ser to Ala substitutions at residues Ser273, Ser282, and Ser302 (i.e., 3SA)], at sarcomere length (SL) 1.9 μm or 2.1μm, prior and following protein kinase A (PKA) treatment. Steady-state force generation measurements revealed a blunting in the length-dependent increase in myofilament Ca^2+^-sensitivity of force generation (pCa_50_) following an increase in SL in 3SA skinned myocardium compared to WT skinned myocardium. Dynamic XB behavior was assessed at submaximal Ca^2+^-activations by imposing an acute rapid stretch of 2% of initial muscle length, and measuring both the magnitudes and rates of resultant phases of force decay due to strain-induced XB detachment and delayed force rise due to recruitment of additional XBs with increased SL (i.e., stretch activation). The magnitude (P2) and rate of XB detachment (*k*_rel_) following stretch was significantly reduced in 3SA skinned myocardium compared to WT skinned myocardium at short and long SL, and prior to and following PKA treatment. Furthermore, the length-dependent acceleration of *k*_rel_ due to decreased SL that was observed in WT skinned myocardium was abolished in 3SA skinned myocardium. PKA treatment accelerated the rate of XB recruitment (*k*_df_) following stretch at both SL's in WT but not in 3SA skinned myocardium. The amplitude of the enhancement in force generation above initial pre-stretch steady-state levels (P3) was not different between WT and 3SA skinned myocardium at any condition measured. However, the magnitude of the entire delayed force phase which can dip below initial pre-stretch steady-state levels (P_df_) was significantly lower in 3SA skinned myocardium under all conditions, in part due to a reduced magnitude of XB detachment (P2) in 3SA skinned myocardium compared to WT skinned myocardium. These findings demonstrate that cMyBP-C phospho-ablation regulates SL- and PKA-mediated effects on XB kinetics in the myocardium, which would be expected to contribute to the regulation of the Frank-Starling mechanism.

## Introduction

Two important physiological mechanisms by which the heart modulates its ability to increase the strength and rate of contraction are length-dependent changes in the contractile function of cardiac muscle (Ter Keurs et al., 1980), and enhanced activation of the β-adrenergic signaling pathway (Kranias and Solaro, 1982). At the whole-heart level, length-dependent activation (LDA) increases ventricular pressure development in response to increased ventricular filling i.e., an increased stretch of the ventricular muscle wall (Allen and Kentish, 1985; De Tombe et al., 2010)—a phenomenon that drives the Frank-Starling Law of the heart (Solaro, 2007). In cardiac myocytes LDA is reflected as an enhancement in force generation in response to an increase in sarcomere length (SL). At the myofilament level, factors that modulate LDA include changes in lattice spacing (Fuchs and Smith, 2001), myofilament responsiveness to [Ca^2+^] (Konhilas et al., 2002; Cazorla et al., 2006), rates of cross-bridge (XB) cycling (Adhikari et al., 2004; Moss et al., 2004; Biesiadecki et al., 2014), and a complex interplay between XB-induced cooperative activation and cooperative deactivation of thin filaments (Hanft et al., 2008).

In addition, increased β-adrenergic signaling in response to increased cardiac workload increase levels of cyclic AMP and protein kinase A (PKA) (Kranias and Solaro, 1982), resulting in increased phosphorylation of proteins involved in excitation-contraction coupling such as phospholamban, L-type Ca^2+^ channels, and ryanodine receptors which contribute to enhanced systolic and diastolic function (Bers, 2002). At the myofilament level, PKA targets key regulatory proteins such as cardiac troponin I (cTnI) (Kentish et al., 2001; Layland et al., 2004), titin (Fukuda and Granzier, 2005), and cardiac myosin binding protein-C (cMyBP-C) (Garvey et al., 1988; Stelzer et al., 2007b; Barefield and Sadayappan, 2010). PKA-mediated phosphorylation of myofilament proteins in turn affects LDA by impacting multiple processes that control the rates of thin filament deactivation and XB cycling (reviewed by Biesiadecki et al., 2014), and the rates of XB recruitment and force generation (reviewed by Moss et al., 2015). In particular, the role of cTnI phosphorylation in modulating LDA has been fairly well investigated; PKA phosphorylation of residues Ser23/24 on cTnI has been shown to decrease myofilament Ca^2+^ sensitivity of force generation (Solaro et al., 1976; Robertson et al., 1982; Fentzke et al., 1999). Recently, it has also been suggested that other phosphorylatable residues on cTnI such as Thr 143 may also play a role in modulating LDA (Tachampa et al., 2007; Wijnker et al., 2014). The effects of PKA phosphorylation (which targets both cTnI and cMyBP-C) on XB kinetics are less clear with some showing a decrease (Hanft and Mcdonald, 2009, 2010), no change (Janssen and De Tombe, 1997; Walker et al., 2011) or an acceleration (Stelzer et al., 2006d; Cheng et al., 2013; Gresham et al., 2014). Furthermore, PKA-mediated phosphorylation led to more pronounced LDA in myocardial preparations lacking the titin's N2B region due to enhanced length-dependent changes in myofilament Ca^2+^ sensitivity and passive tension—suggesting a role for titin in modulating LDA (Lee et al., 2013). It has been suggested that cMyBP-C phosphorylation also plays a role in modulation of length-dependent changes in steady-state contractile function (Cazorla et al., 2006; Chen et al., 2010; Kumar et al., 2015), however, the contribution of cMyBP-C phosphorylation in modulating length-dependent changes in myofilament XB kinetics is still unknown.

Results from earlier investigations provide strong evidence for a significant role for cMyBP-C phosphorylation in modulating length-dependent changes in XB kinetics. Specifically, cMyBP-C phosphorylation has been shown to increase the proximity between myosin heads and actin (Colson et al., 2008), and also regulates key aspects of XB behavior that are known to impact LDA (Hanft et al., 2008) such as the rates of XB detachment and XB recruitment during Ca^2+^ activation (Stelzer et al., 2006c; Lecarpentier et al., 2008; Tong et al., 2008; Coulton and Stelzer, 2012; Michalek et al., 2013; Wang et al., 2014). Furthermore, recent studies show that increased cMyBP-C phosphorylation augments force generation and the amplitude of the cardiac twitches in intact cardiac preparations (Tong et al., 2015), and modulates *in vivo* contractile and hemodynamic properties by enhancing the systolic pressure development and diastolic pressure relaxation (Rosas et al., 2015; Gresham and Stelzer, 2016). We recently demonstrated that transgenic (TG) mice expressing non-phosphorylatable cMyBP-C containing Ser to Ala substitutions at residues Ser273, Ser282, and Ser302 (i.e., 3SA, Tong et al., 2008; Gresham and Stelzer, 2016), displayed depressed accelerations of *in vivo* left-ventricular pressure development and pressure relaxation in response to acute β-agonist infusion, demonstrating that cMyBP-C phosphorylation is a primary mediator of the cardiac contractile response to increased β-adrenergic stimulation (Gresham and Stelzer, 2016).

Therefore, to define the precise molecular mechanisms of cMyBP-C phosphorylation in modulating length-dependent changes in contractile function, we performed mechanical experiments in skinned myocardium isolated from WT and 3SA hearts at variable SL (1.9 and 2.1 μm), prior to and following PKA treatment. We utilized stretch-activation experiments to probe dynamic XB behavior because stretch-activation, i.e., the delayed force development resulting from the stretch of the ventricular wall, has been proposed to be an intrinsic length-sensing mechanism that plays a vital role in mediating LDA in myocardial contraction on a beat-to-beat basis (Campbell and Chandra, 2006; Stelzer and Moss, 2006). Specifically, imposing a sudden rapid stretch on a muscle fiber during steady-state isometric contraction elicits a multiphase force response which exhibits an immediate increase in force that is proportional to the magnitude of the imposed stretch. This initial rise in force is due to the distortion of the elastic regions of the bound XBs which then rapidly decays as the distorted XBs detach and repopulate into the nondistorted state (Davis and Rodgers, 1995; Piazzesi et al., 1997). This force decay phase is followed by a slow, gradual force redevelopment phase due to stretch-induced recruitment of additional XBs into the force-producing state (Lombardi et al., 1995; Dobbie et al., 1998) which is referred to as the stretch-activation response (for alternative interpretations of the stretch-activation phases and how they relate to various XB models, refer to, Abbott and Steiger, 1977; Ford et al., 1977; Lombardi et al., 1995; Piazzesi et al., 1997; Davis and Epstein, 2003; Kawai and Halvorson, 2007). It was long recognized that cardiac muscle exhibits a prominent stretch-activation response (Steiger, 1971), a phenomenon that contributes to significant enhancement in force generation during the systolic ejection (Vemuri et al., 1999; Davis et al., 2001), and also contributes to the steepness of the length-tension relationship in cardiac muscle (Allen and Kentish, 1985; Campbell and Chandra, 2006). The phenomenon of stretch-activation is most pronounced at low levels of Ca^2+^ activation, because under these conditions relatively few XBs are strongly-bound to the thin filament, and the majority of the actin binding sites are available for recruitment and binding of additional XBs (Stelzer et al., 2006c). Thus, stretch-activation consists of both the initial binding of the XBs to the thin filament and the subsequent cooperative recruitment of additional unbound XBs, thereby promoting the propagation of activation of neighboring thin filament regulatory subunits in response to a sudden stretch in muscle length.

Our results demonstrate that cMyBP-C phospho-ablation significantly slows the rate of XB detachment (*k*_rel_) in response to a rapid acute stretch, and also abolishes the acceleration in *k*_rel_ due to decreased SL and PKA treatment that is observed in WT skinned myocardium. Furthermore, our data show that skinned myocardium isolated from 3SA hearts displays a significantly reduced magnitude of stretch-induced XB recruitment at either SL, or following PKA treatment compared to WT skinned myocardium, suggesting that cMyBP-C phosphorylation facilitates length-dependent changes in cooperative XB recruitment and cycling. Thus, our data show that cMyBP-C phosphorylation is a requisite for eliciting normal length-dependent modulation of XB kinetics in the cardiac sarcomere, and would be expected to contribute to the LDA and consequent enhancement in the Frank-starling mechanism in conditions of increased sympathetic drive (Hanft and Mcdonald, 2009).

## Materials and methods

### Ethical approval and animal treatment protocols

All experiments described in this study were performed as outlined in the *Guide for the Care and Use of Laboratory Animals* (NIH Publication No. 85–23, Revised 1996), and were conducted in accordance with the guidelines of the Institutional Animal Care and Use Committee at the Case Western Reserve University. Male and female wild-type (WT) and transgenic (TG) mice expressing non-phosphorylatable cMyBP-C containing serine (Ser) to alanine (Ala) substitutions at residues Ser273, Ser282, and Ser302 (i.e., 3SA) on a cMyBP-C null background (Tong et al., 2008; Gresham and Stelzer, 2016), aged 3–6 months (SV/129 strain) were used for the experiments.

### Determination of phosphorylation status of cMyBP-C and other sarcomeric proteins in WT and 3SA myocardial samples

Determination of myofilament protein phosphorylation status was done by Western blot and Pro-Q Diamond phosphoprotein stain (Life Technologies) as described previously (Gresham et al., 2014; Mamidi et al., 2014). Isolation of cardiac myofibrils from frozen mouse ventricles was performed as described previously (Cheng et al., 2013; Gresham et al., 2014; Mamidi et al., 2015). Frozen mouse ventricular tissue was thawed and homogenized in fresh relaxing solution on the day of the experiment. Myofibrils were chemically skinned for 15 min using 1% Triton X-100 on a mechanical rocker plate, centrifuged, and then resuspended in fresh relaxing solution containing protease and phosphatase inhibitors (PhosSTOP and cOmplete ULTRA Tablets; Roche Applied Science, Indianapolis, IN, USA), and stored on ice until further use. All solutions were brought to room temperature (22°C) for 10 min before initiating the PKA phosphorylation reaction. One hundred micro grams of WT and 3SA myofibrils were incubated with the catalytic subunit of bovine PKA (Sigma-Aldrich, St Louis, MO, USA) to a final concentration of 0.15 U PKA/μg myofibrils for 1 h at 30°C (Gresham et al., 2014). Control myofibrils were incubated under the same conditions without PKA. Laemli buffer was added to stop the reaction and samples were heated at 90°C for 5 min and stored at −20°C until the gels were run. For Western blot, 5 μg of solubilized myofibrils were loaded onto a 4–20% Tris-glycine gel (Lonza, Rockland, ME, USA) and electrophoretically separated at 180V for 70 min. Proteins were transferred to PVDF membranes and incubated overnight with one of the following primary antibodies: total TnI (Cell Signaling Technology), TnI phospho-serine 23 and 24 (detects phosphorylation of Ser23 and Ser24 of TnI; Cell Signaling), total cMyBP-C (Santa Cruz Biotechnology), cMyBP-C phospho-serine 273, 282, or 302 (detects phosphorylation of Ser273, S282, or Ser302 of cMyBP-C; 21st Century Biochemicals), or HSC70 as a loading control (Santa Cruz Biotechnology). Membranes were then incubated with appropriate secondary antibodies and imaged. For estimating the total myofilament protein phosphorylation, 2.5 μg of myofibrils were separated at 180V for 85 min, fixed, and then stained with Pro-Q phosphostain and imaged using a Typhoon gel scanner. Pro-Q gels were counterstained with coomassie blue to determine the total protein loaded. Densitometric scanning of the stained gels was done using Image J software (U.S. National Institutes of Health, Bethesda, MD, USA; Gresham et al., 2014).

### Preparation of skinned myocardial preparations and Ca^2+^ solutions for mechanical experiments

Skinned myocardium was prepared as described previously (Cheng et al., 2013; Gresham et al., 2014). In brief, ventricular tissue was homogenized in a relaxing solution followed by chemical-skinning for 1 h using 1% Triton-X 100 (Thermo Scientific, Rockford, IL). Multicellular ventricular preparations measuring ~100 μm in width and ~400 μm in length were chosen for the experiments. The composition of various Ca^2+^ activation solutions used for the experiments was calculated using a computer program (Fabiato, 1988) and established stability constants (Godt and Lindley, 1982). All solutions contained the following (in mM): 14.5 creatine phosphate, 7 EGTA, and 20 Imidazole. The maximal activating solution (pCa 4.5; pCa = -log [Ca^2+^]_free_) also contained 65.45 KCl, 7.01 CaCl_2_; 5.27 MgCl_2_, 4.81 ATP, while the relaxing solution (pCa 9.0) contained 72.45 KCl, 0.02 CaCl_2_; 5.42 MgCl_2_, 4.76 ATP. The pH of the Ca^2+^ solutions was set to 7.0 and the ionic strength was 180 mM. A range of pCa solutions (pCa 6.3 to 5.5), containing varying amounts of [Ca^2+^]_free_, were then prepared by mixing appropriate volumes of pCa 9.0 and 4.5 stock solutions, and all the experiments were carried out at 23°C.

### Experimental apparatus for measurement of contractile properties in skinned myocardium

Chemically-skinned multicellular ventricular preparations were mounted between a motor arm (312C; Aurora Scientific Inc., Aurora, Ontario, Canada) and a force transducer (403A; Aurora Scientific Inc.) as described previously (Merkulov et al., 2012; Cheng et al., 2013). Changes in the motor position and signals from the force transducer were sampled at 2000 Hz using sarcomere length (SL) control software program (Campbell and Moss, 2003). For all mechanical measurements, SL of the ventricular preparations was set to either 1.9 μm (short SL) or 2.1 μm (long SL) (Desjardins et al., 2012; Cheng et al., 2013). Force-pCa relationships were generated by incubating the skinned myocardial preparations in a range of pCa solutions (i.e., pCa 6.3 to 4.5). The apparent cooperativity of force development was estimated from the steepness of Hill plot transformation of the force-pCa relationships (Mamidi et al., 2014). The force-pCa data were fit using the equation P/P_o_ = [Ca^2+^]^*nH*^/(*k*^*nH*^ + [Ca^2+^]^*nH*^), where *n*_H_ is the Hill coefficient and *k* is the pCa required to produce half-maximal activation (i.e., pCa_50_; Gresham et al., 2014).

### Measurement of the rate of force redevelopment (*k*_tr_)

*k*_*tr*_ was measured in WT and 3SA skinned myocardium to assess XB transitions from both the weakly- to strongly-bound states and from the strongly- to weakly-bound states (Brenner and Eisenberg, 1986; Campbell, 1997). A mechanical slack-restretch protocol was used to measure *k*_tr_ in the Ca^2+^-activated myocardial preparations as described previously (Stelzer et al., 2006b; Chen et al., 2010; Cheng et al., 2013). Skinned myocardial preparations were transferred from relaxing (pCa 9.0) to Ca^2+^ activating solutions (pCa ranging from 6.1 to 5.8) yielding ~35% of maximal activation level, and when the myocardial preparations attained a steady-state isometric force, they were rapidly slackened by 20% of their original muscle length and were held constant for 10 ms. The slackening was followed by a brief period of unloaded shortening resulting in a rapid force decline due to the detachment of the strongly-bound XBs. The myocardial preparations were then rapidly stretched back to their original length and the time course of force redevelopment was measured. *k*_tr_ was estimated by linear transformation of the half-time of force redevelopment, i.e., *k*_tr_ = 0.693/t_1∕2_, where t_1∕2_ is the time (in milliseconds) taken to reach the half maximal force of the *k*_tr_ trace as described previously (Merkulov et al., 2012; Cheng et al., 2013; Mamidi et al., 2015). Baseline force was considered the point on the *k*_tr_ trace where force begins to redevelop following the slack-restretch maneuver, and peak force development was considered the point in the *k*_tr_ trace where force plateaus and reaches a steady-state level.

### Stretch activation experiments to determine dynamic XB contractile parameters

**Figure 1:**
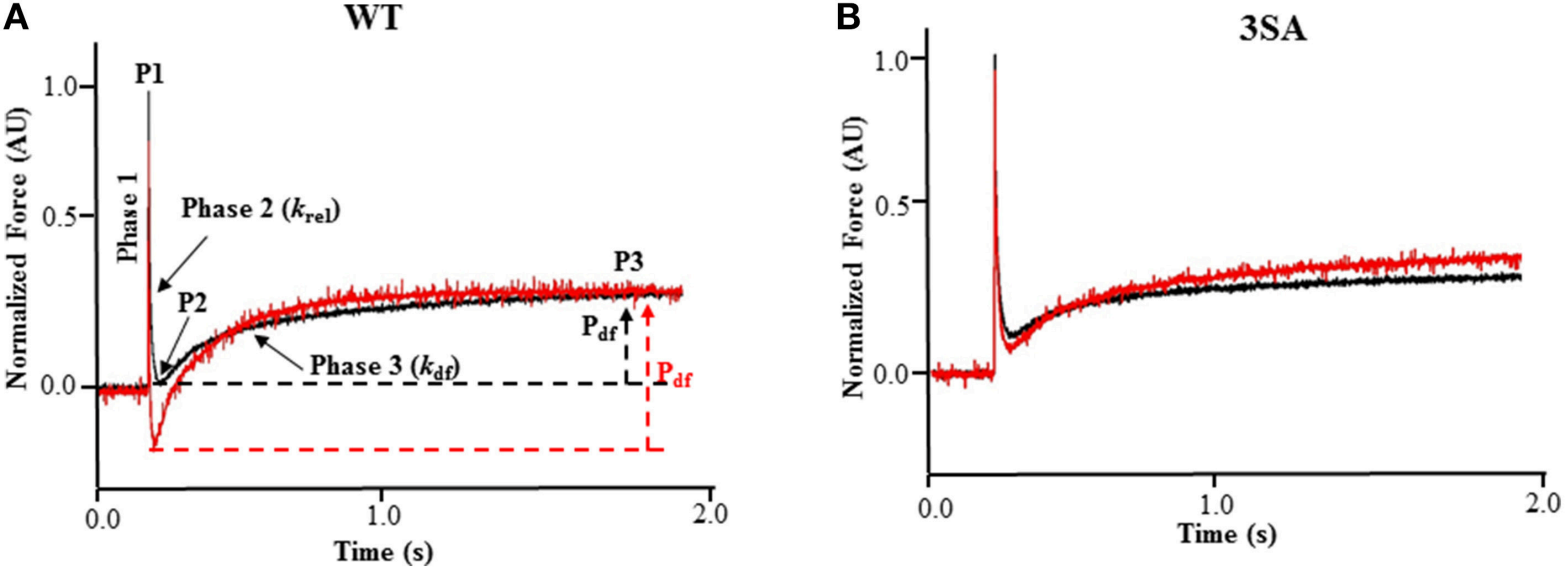
**Effect of PKA treatment on the stretch activation responses in WT and 3SA skinned myocardium**. Traces of representative force responses elicited at ~35% of maximal Ca^2+^ activation level by a sudden 2% stretch in muscle length (ML) in isometrically-contracting **(A)** WT and **(B)** cMyBP-C phospho-ablated (i.e., 3SA) myocardial preparations prior to (black) and following incubation with PKA (red). In **(A)**, highlighted are the important phases of the force transients and the various stretch activation parameters that are derived from the elicited force response (see Materials and Methods). Phase 1 represents the immediate increase in force in response to the sudden increase in ML. P1 is the magnitude of the immediate force response and is measured from the pre-stretch isometric steady-state force to the peak of phase 1, and it represents the magnitude of XB stiffness. Phase 2 represents the rapid decay in force with a dynamic rate constant *k*_rel_ and is an index of the rate of XB detachment. P2 represents the minimum force attained at the end of Phase 2 of the stretch-activation response and is an index of the magnitude of XB detachment. Phase 3 represents the delayed force development with a dynamic rate constant *k*_df_ and is an index of the rate of XB recruitment. P3 represents the new steady-state force attained in response to the imposed stretch in muscle length and is an index of force enhancement above initial pre-stretch isometric levels. P_df_ represents the amplitude of the delayed force development and is an index of the overall number of XBs being recruited into the force-bearing state in response to a sudden 2% stretch in ML i.e., it represents the magnitude of XB recruitment. PKA treatment significantly accelerated both *k*_rel_ and *k*_df_ in WT skinned myocardium but not in 3SA skinned myocardium. AU, arbitrary units.

Stretch activation experiments were performed as described earlier (Gollapudi et al., 2012; Cheng et al., 2013; Gresham et al., 2014; Michael et al., 2014). Skinned myocardial preparations were bathed in Ca^2+^ solutions yielding steady-state forces of ~35% of maximal, and once the myocardial preparations reached a steady-state force, they were then rapidly stretched by 2% of their initial muscle length, held at the new length for 5 s, and were then returned back to their initial muscle length. In our experiments, high speed stretches (completed ~ in 2 ms) were imposed so as to minimize the changes in XB populations during the time of the imposed stretch in muscle length, so that the stretch activation response observed was likely due to the elastic properties of the XBs bound to actin prior to the stretch (Stelzer et al., 2006c). The characteristic features of the stretch activation responses/tension transients in cardiac muscle have been described in detail elsewhere (Stelzer and Moss, 2006; Ford et al., 2010), and the stretch activation parameters measured are shown in Figure 1. Different phases of the tension transients elicited in response to step increases in length were then analyzed individually as done previously to gain insights into XB mechanics (Stelzer et al., 2006a,c,d, 2007b).

In brief, a sudden 2% stretch of muscle length causes an instantaneous rise in force (P1), which is a result of strain of strongly-bound XBs (Phase 1) and denotes XB stiffness (Stelzer and Moss, 2006; Mamidi et al., 2014). The force then quickly decays (Phase 2) due to a rapid detachment of the strained XBs which equilibrate into a non-force generating state, with a rate constant *k*_rel_, an index of XB detachment. The minimum force attained at the end of Phase 2 of the stretch activation response is denoted by P2, and represents the magnitude of XB detachment following stretch (Stelzer et al., 2006c). The amplitude P2 (i.e., the minimum force attained at the lowest point of the Phase 2 force decay, i.e., the nadir, just prior to the commencement of Phase 3 delayed force development) can decline further than the isometric pre-stretch force resulting in negative values, especially following PKA treatment, which is likely due to an acceleration in the rate of XB detachment in phase 2 (Stelzer et al., 2006d). Following Phase 2, the preparations exhibit a gradual rise in force development (Phase 3), with a rate constant *k*_df_, due to stretch-induced recruitment of additional XBs into the force-generating state, and the amplitude of Phase 3 is an index of the magnitude of XB recruitment (Stelzer et al., 2006c; Gresham et al., 2014). The new peak of the steady-state force, which is higher than the initial steady-state force (i.e., the pre-stretch force), attained in response to the increase in muscle length is indicated by P3 (Figure 1A). The sum of all XBs recruited by a sudden 2% stretch in muscle length in an isometrically-contracting myocardial preparation can be assessed by measuring the amplitude of the delayed force response of Phase 3 (Stelzer et al., 2006c). When P2 values are negative, the trough-to-peak amplitude of Phase 3 (i.e., P_df_) exceeds that of P3, and therefore, in these cases P_df_ represents the sum of all XB recruitment in Phase 3 due to stretch (Stelzer et al., 2006d). As the level of Ca^2+^ activation increases, P3 and P_df_ decrease because a greater number of XBs are already bound to actin prior to the stretch and fewer XBs are now available in the non-force generating pool for recruitment into the force-generating pool upon a stretch in muscle length (Stelzer and Moss, 2006).

Stretch activation amplitudes were measured manually by analyzing the different phases of the stretch activation transients. The different stretch activation amplitudes were normalized to pre-stretch Ca^2+^-activated force to facilitate comparisons between preparations that develop different amounts of absolute force, and the Ca^2+^-activation levels as done before (Stelzer et al., 2006d, 2007b). Thus, the amplitudes of all parameters measured are expressed as a fraction of the total pre-stretch forces (**Table 2**; Stelzer et al., 2007b). Stretch activation amplitudes were measured individually by manually fitting different phases of the tension transients (Stelzer et al., 2006d, 2007b) as follows and are shown in Figure 1A.

P1: measured from the pre-stretch steady-state force to the peak of phase 1
P2: measured from the pre-stretch steady-state force to the minimum force value attained at the end of phase 2.
P3: measured from the pre-stretch steady-state force to the peak force value of the delayed force attained in phase 3.
P_df_: is the difference between P3 and P2

*k*_rel_ was measured by fitting a single exponential to the time course of force decay using the formula: *y* = a (-1+ exp (-k_1_×x)) where “a” is the amplitude of the single exponential phase and k_1_ is the rate constant of the force decay.

Similar to *k*_tr_ measurements, *k*_df_ was estimated by linear transformation of the half-time of force redevelopment, i.e., *k*_*df*_ = 0.693/t_1∕2_, where t_1∕2_ is the time (in milliseconds) taken from the nadir (i.e., the point of force reuptake at the end of phase 2) to achievement of the half maximal force of phase 3 of the force response shown in Figure 1, where maximal force is indicated by a plateau region in phase 3 i.e., P3. Thus, *k*_df_ represents the rate of recruitment of all XBs that give rise to the delayed force transient following stretch (i.e., P_df_).

Following incubation with PKA, the stretch activation experiments were repeated. Because PKA treatment decreases the myofilament Ca^2+^ sensitivity of force generation, we used a pCa solution with slightly higher [Ca^2+^]_free_ to match the activation levels prior to PKA treatment (Stelzer et al., 2006d, 2007b).

### Data analysis

All data are reported as mean ± SEM. Steady-state and dynamic contractile parameters were analyzed using a three-way analysis of variance (ANOVA) by fitting linear models of three factors using the R statistical program (R Core Team, 2013). One factor in this analysis was cMyBP-C phosphorylation (WT or 3SA), the second was PKA treatment (-PKA or +PKA), and the third was SL (SL 1.9 or 2.1 μm). Using this analysis, we assessed the three-way and the two-way interaction effects. When the interaction effects were not significant, we interpreted the main effects due to cMyBP-C phosphorylation, PKA, or SL. To probe the cause for the interaction or main effects, post-hoc multiple pairwise comparisons were made using Fisher's Least Significant Difference (Fisher's LSD) method as done previously (Ford et al., 2012). The criterion for statistical significance was set at *P* < 0.05, and the asterisks in figures and tables represent statistical significance using post-hoc Fisher's LSD tests.

## Results

### Effect of cMyBP-C phospho-ablation on the isoform expression and phosphorylation status of sarcomeric proteins

**Figure 2:**
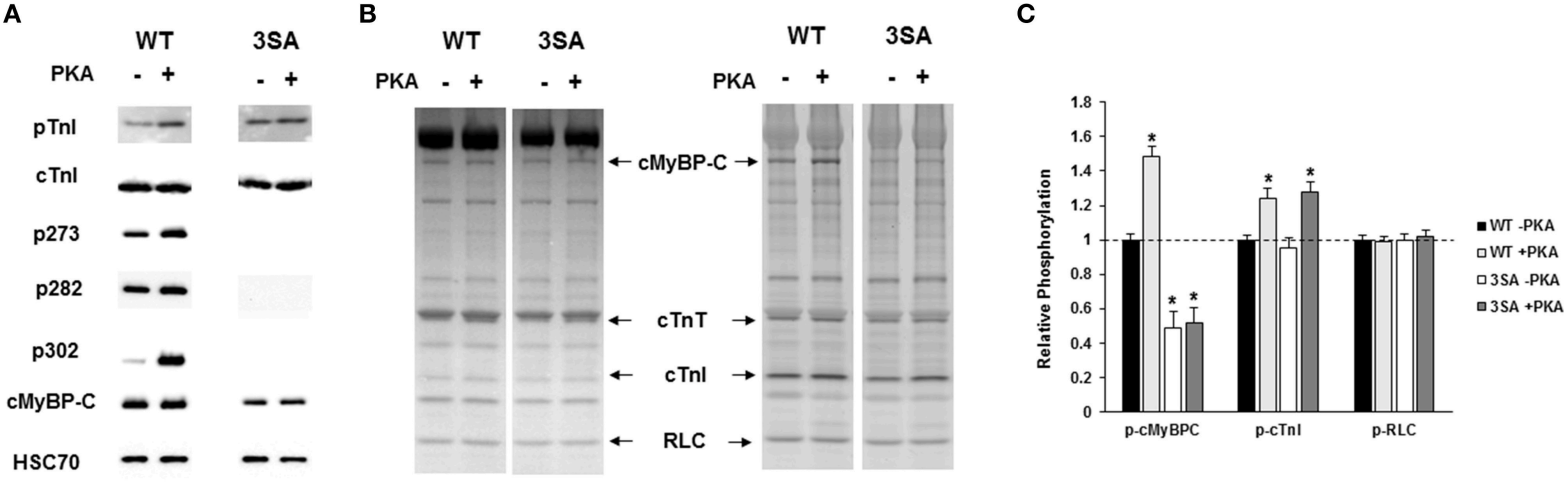
**Western Blot and Pro-Q analysis to assess the phosphorylation status of myofilament filament proteins in WT and 3SA myocardium. (A)** Western blots showing cMyBP-C and cTnI phosphorylation prior to and following PKA treatment in WT and 3SA heart samples. cTnI phosphorylation at residues Ser23/24 was similar between WT and 3SA samples under basal conditions (-PKA) and also following PKA treatment. cMyBP-C phosphorylation at residues Ser273, Ser282, and Ser302 was absent in 3SA heart samples, and their phosphorylation levels were enhanced in the WT samples following PKA incubation. **(B)** Representative Pro-Q Diamond-stained (right) and Coomassie-stained (left) SDS gels showing the expression and phosphorylation status of myofilament proteins prior to and following PKA treatment in WT and 3SA heart samples. **(C)** Quantification of protein phosphorylation in WT and 3SA hearts as determined by Pro-Q Diamond staining. The intensity of the phosphorylation signal was normalized to the intensity of the total protein signal and the untreated WT myofibril protein phosphorylation was set to 1 as done in our previous studies (Gresham et al., 2014; Mamidi et al., 2015). cMyBP-C phosphorylation was enhanced following PKA treatment in WT samples but not in 3SA samples. No differences in phosphorylation of other myofilament proteins were observed between WT and 3SA samples under basal conditions and following PKA treatment. cTnI phosphorylation was significantly enhanced following PKA treatment in both WT and 3SA samples. Basal phosphorylation levels of cMyBP-C (no PKA) observed in 3SA hearts was similar to that reported in an earlier study (Tong et al., 2008). Myofibrils were isolated from 5 to 6 hearts for quantification of protein phosphorylation in WT and 3SA groups. Asterisks in **(C)** indicate statistical differences when compared to the corresponding pre PKA WT group. WT, wild-type; cMyBP-C, cardiac myosin binding protein-C; 3SA, non-phosphorylatable cMyBP-C; cTnT, cardiac troponin T; cTnI, cardiac troponin I; RLC, regulatory light chain.

To determine the impact of TG expression of 3SA on isoform expression and phosphorylation status of key regulatory sarcomeric proteins, cardiac samples isolated from WT and 3SA heart were analyzed by Western blot and Pro-Q Diamond phospho-stain analysis (Figure 2). The myocardial expression of 3SA cMyBP-C was determined to be ~73 ± 8% of the cMyBP-C content present in the WT samples, as reported earlier (Tong et al., 2008; Gresham and Stelzer, 2016). Western blot analysis (Figure 2A) shows no differences in the phosphorylation levels of cTnI PKA residues Ser23/24 between WT and 3SA samples, but as expected the phosphorylation of cMyBP-C at residues Ser273, Ser282, and Ser302 was absent in 3SA samples confirming phospho-ablation of all three PKA-phosphorylatable Ser residues in the M-domain of cMyBP-C (Gresham and Stelzer, 2016).

Ventricular samples from WT and 3SA hearts were also stained with Pro-Q Diamond to assess the impact of 3SA expression on the phosphorylation status of other myofilament regulatory proteins (Figures 2B,C). Our results show that the expression and phosphorylation levels of sarcomeric proteins such as troponin T and regulatory light chain were not significantly different between WT and 3SA samples (Figures 2B,C).

To determine the effect of cMyBP-C phospho-ablation on PKA-mediated phosphorylation of myofilament proteins, WT and 3SA samples were analyzed by Western blot and Pro-Q Diamond staining following incubation with PKA for 1 h at 30°C (Tong et al., 2008; Gresham et al., 2014). PKA treatment enhanced phosphorylation of cTnI to a similar extent in both WT and 3SA samples (Figure 2C). In contrast, PKA-mediated enhancement of phosphorylation of cMyBP-C was evident in WT samples but not in 3SA samples (Figures 2A–C), demonstrating that PKA-mediated phosphorylation of cMyBP-C was abolished in 3SA samples.

### Effect of cMyBP-C phospho-ablation on length-dependent changes in steady-state force generation

To assess the impact of cMyBP-C phospho-ablation on SL-dependent changes in thin-filament activation, steady-state Ca^2+^-activated force generation was measured at SL 1.9 and 2.1 μm in WT and 3SA myocardial preparations (values are shown in Table 1). We recently demonstrated (Gresham et al., 2014) that steady-state and dynamic contractile parameters of skinned myocardium isolated from WT hearts and TG hearts expressing un-mutated cMyBP-C on a null cMyBP-C background (Tong et al., 2008; i.e., TG^WT^), are not different at baseline and following PKA incubation. Thus, in this study we performed mechanical measurements on skinned myocardial preparations isolated from WT and 3SA hearts.

**Table 1:** **Steady-state contractile parameters measured in WT and 3SA skinned myocardium**.

**Group**	**pCa_50_**	***n*_H_**	**F_max_(mN/mm^2^)**	**F_min_(mN/mm^2^)**
**SL 1.9 μm**				
WT (−PKA)	5.79 ± 0.02 (13)	3.49 ± 0.18 (13)	12.21 ± 1.44 (13)	0.33 ± 0.04 (13)
WT (+PKA)	5.74 ± 0.01 (10)^*^	3.38 ± 0.43 (10)	11.25 ± 1.38 (10)	0.43 ± 0.08 (10)
3SA (−PKA)	5.83 ± 0.01 (11)	3.59 ± 0.20 (11)	12.98 ± 1.70 (11)	0.45 ± 0.09 (11)
3SA (+PKA)	5.76 ± 0.03 (11)^*^	3.05 ± 0.19 (11)	11.24 ± 1.41 (11)	0.46 ± 0.10 (11)
**SL 2.1 μm**				
WT (−PKA)	5.86 ± 0.01 (17)^†^	2.49 ± 0.12 (17)^†^	18.99 ± 1.73 (17)^†^	1.16 ± 0.10 (17)^†^
WT (+PKA)	5.75 ± 0.01 (12)^*^	2.86 ± 0.16 (12)	17.83 ± 1.79 (12)^†^	1.12 ± 0.16 (12)^†^
3SA (−PKA)	5.88 ± 0.01 (11)^†^	2.78 ± 0.26 (11)^†^	20.22 ± 2.70 (11)^†^	1.50 ± 0.20 (11)^†^
3SA (+PKA)	5.78 ± 0.01 (12)^*^	2.80 ± 0.20 (12)	20.22 ± 3.65 (12)^†^	1.15 ± 0.21 (12)^†^

*pCa_50_, myofilament Ca^2+^ sensitivity; n_H_, cooperativity of force production; F_max_, maximal Ca^2+^ activated force measured at pCa 4.5; F_min_, Ca^2+^ independent force measured at pCa 9.0. A minimum of 4 hearts per group were used, and the number of preparations for each group is presented in brackets. Values are expressed as mean ± S.E.M*.

* *Significantly different compared to the corresponding (-PKA) group at the same SL*.

† *Significantly different compared to the corresponding group at short SL; asterisks indicate P < 0.05*.

**Figure 3:**
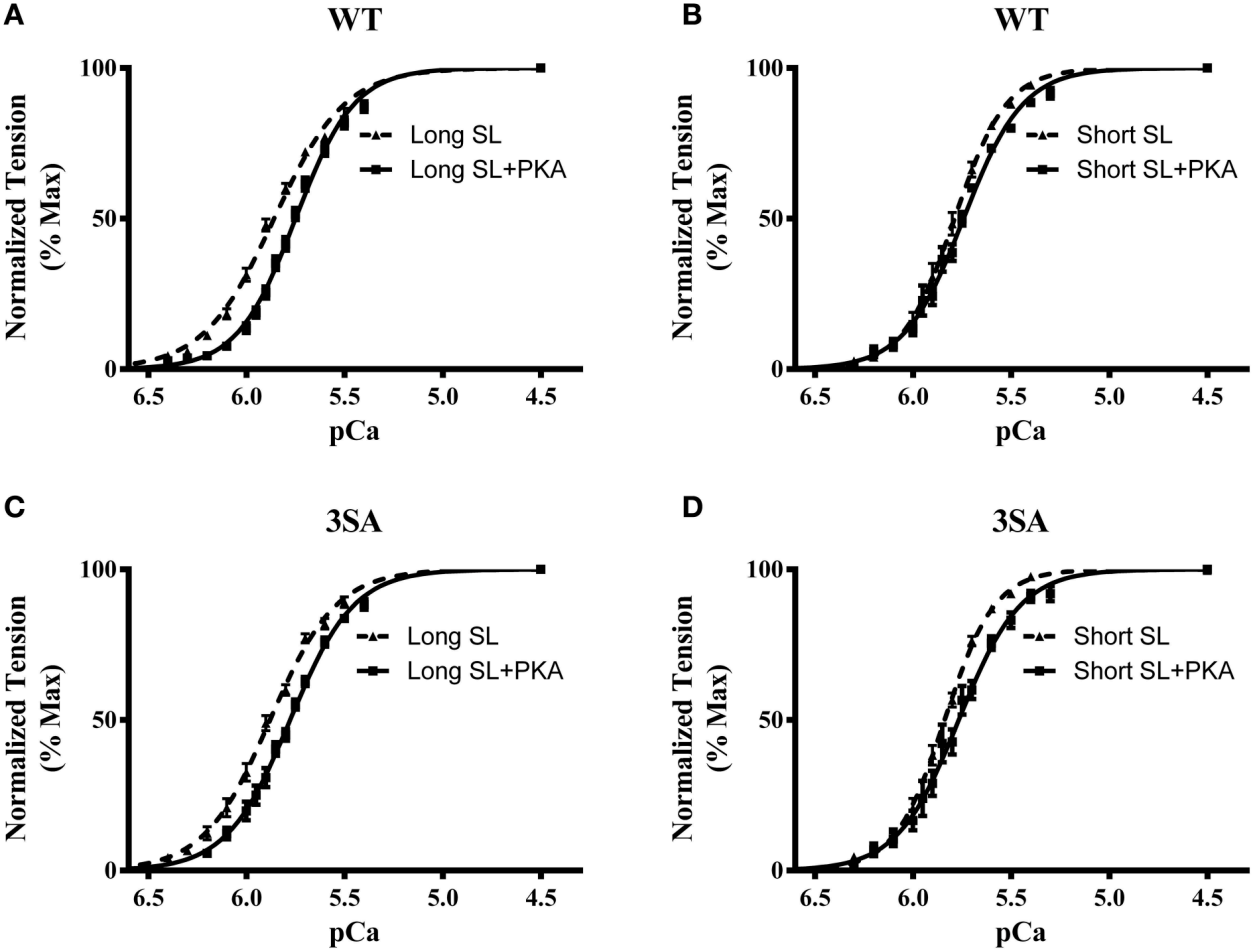
**Effect of PKA treatment on myofilament Ca^**2+**^ sensitivity (pCa_**50**_) in WT and 3SA skinned myocardium**. Force-pCa relationships were constructed by plotting normalized forces generated by incubating the myocardial preparations in a range of pCa prior to and following PKA treatment. Effect of PKA treatment on the force-pCa relationships in WT preparations at **(A)** long SL, and **(B)** short SL. Effect of PKA treatment on the force-pCa relationships in 3SA preparations at **(C)** long SL, and **(D)** short SL. PKA treatment resulted in a significant right-ward shift (decrease in pCa_50_) in the force-pCa relationships in all the groups (values are shown in Table 1). The number of preparations used for each group are shown in Table 1. A minimum of 4 hearts per group were used with multiple preparations from each heart.

Increasing SL from 1.9 to 2.1 μm enhanced force generation in WT and 3SA skinned myocardium to a similar extent as demonstrated by an increase in both maximal force generation (F_max_, measured at pCa4.5) and minimal force generation (F_min_, measured at pCa 9.0; Table 1). pCa_50_ was also significantly increased at SL 2.1 μm compared to 1.9 μm in both WT and 3SA groups (Figure 3; Table 1), indicating that myofilament Ca^2+^ sensitivity was increased by increasing SL. We found a main effect of cMyBP-C phosphorylation on length-dependent changes in pCa_50_ (Table 1), which was likely due to the less pronounced length-dependent increase in pCa_50_ in the 3SA group under basal conditions (Table 1). PKA treatment significantly reduced pCa_50_ at both long and short SL in 3SA and WT myocardium. Our results also showed that length-dependent changes in pCa_50_ observed in WT and 3SA groups under basal conditions were no longer apparent after PKA treatment (Table 1), causing a significant SL-PKA interaction effect. Cooperativity of force generation, *n*_H_, significantly increased at short SL by ~40% and by ~29% when compared to long SL in the WT and 3SA groups, respectively. Furthermore, PKA treatment did not impact *n*_H_ at both SL's in WT and 3SA groups (Table 1).

### Effect of cMyBP-C phospho-ablation on length-dependent changes in the rate of XB detachment (*k*_rel_)

**Figure 4:**
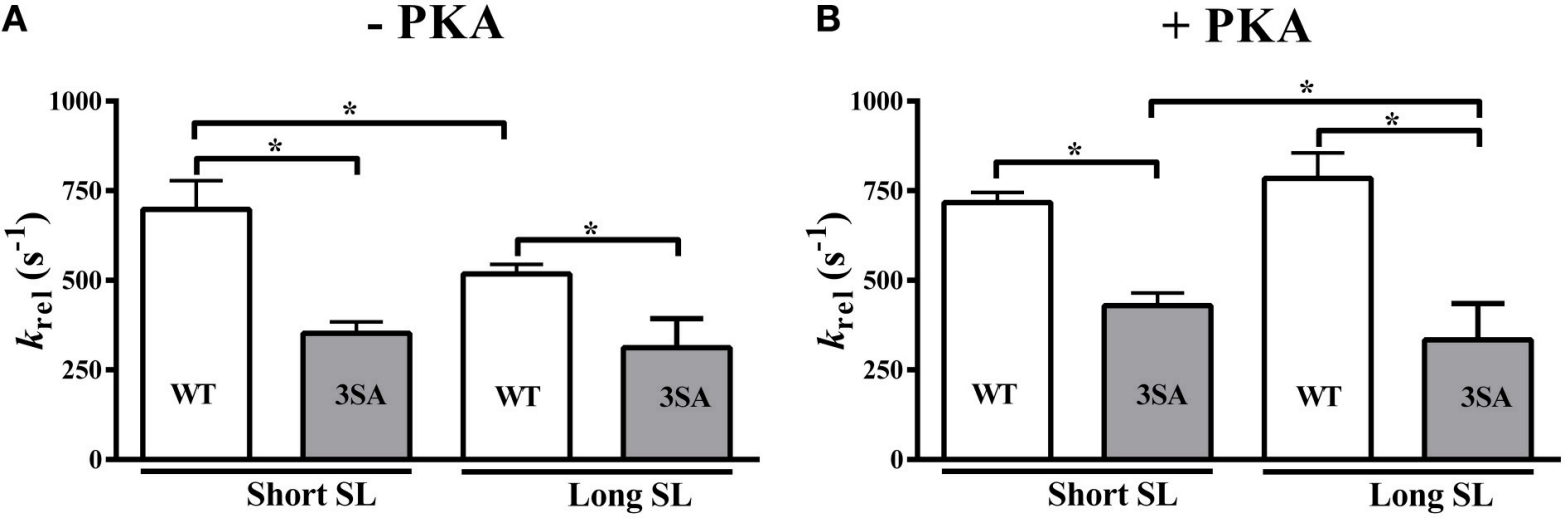
**Effect of cMyBP-C phospho-ablation on SL- and PKA-dependent changes in the rate of XB detachment (***k***_**rel**_)**. Isometrically-contracting myocardial preparations were subjected to a sudden 2% stretch in muscle length and the elicited force responses at ~35% of maximal Ca^2+^ activation level were used to measure **(A)** *k*_rel_ under basal conditions (-PKA) and **(B)** *k*_rel_ following PKA treatment at short and long SL's in WT and 3SA groups. WT preparations displayed significant accelerations in *k*_rel_at short SL compared to long SL under basal conditions, however, no acceleration in *k*_rel_ was observed in 3SA preparations—indicating that SL-dependent changes in XB detachment are abolished in cMyBP-C phospho-ablated skinned myocardium. Furthermore, *k*_rel_was significantly slower in the 3SA preparations compared to WT preparations at both short and long SL's under basal conditions and following PKA treatment—indicating that the rate of XB detachment was significantly slowed in cMyBP-C phospho-ablated skinned myocardium. Values are expressed as mean ± S.E.M. The number of preparations used for each group are shown in Table 2. A minimum of 3 hearts per group were used with multiple preparations from each heart. ^*^P < 0.05.

Our analysis showed that cMyBP-C phosphorylation influences how PKA modulates SL-dependent changes in *k*_rel_(three-way interaction effect). Decreasing SL from 2.1 to 1.9 μm significantly accelerated *k*_rel_ by ~35% in WT myocardium but no effect on *k*_rel_ was observed in 3SA myocardium, suggesting an abolished length-dependent modulation of *k*_rel_ (Table 2; Figure 4A). In addition, PKA treatment significantly accelerated *k*_rel_ by ~52% at long SL in WT myocardium but not in 3SA myocardium. PKA-mediated accelerations in *k*_rel_ were not observed at short SL in either the WT or the 3SA group (Table 2), perhaps suggesting that in these conditions, XB detachment may have approached its maximal rate. The three-way interaction effect on *k*_rel_ was due to the fact that the acceleration in *k*_rel_ observed at short SL in the WT group under basal conditions was absent in the 3SA group (Table 2; Figure 4A).

**Table 2:** **Dynamic stretch-activation parameters measured in WT and 3SA skinned myocardium**.

**Group**	**pCa**	**P1 (P1/P_0_)**	**P2 (P2/P_0_)**	**P3 (P3/P_0_)**	**P_df_ (P_df_/P_0_)**	***k*_tr_(s^−1^)**	***k*_rel_(s^−1^)**	***k*_df_(s^−1^)**
**SL 1.9 μm**								
WT (-PKA)	5.89±0.01(13)	0.492±0.011(13)	−0.004±0.010(13)	0.142±0.006(13)	0.146±0.015(13)	8.03±0.76(13)	698.15±76.35(13)	10.68±1.11(13)
WT (+PKA)	5.79±0.01(9)^†^	0.425±0.012(9)^†^	−0.051±0.007(9)^†^	0.148±0.012(9)	0.199±0.014(9)^†^	10.84±0.75(9)^†^	716.73±28.97(9)	15.41±0.64(9)^†^
3SA (−PKA)	5.87±0.02(12)	0.466±0.019(12)	0.059±0.008(12)^¶^	0.128±0.008(12)	0.069±0.009(12)^¶^	9.28±1.12(12)	352.44±31.58(12)^¶^	10.57±0.97(12)
3SA (+PKA)	5.79±0.01(8)^†^	0.443±0.016(8)	0.060±0.007(8)^€^	0.147±0.010(8)	0.087±0.014(8)^€^	10.18±1.21(8)	429.28±35.54(8)^€^	9.98±0.81(8)^€^
**SL 2.1 μm**								
WT (−PKA)	6.1±0.00(19)^*^	0.583±0.020(19)^*^	0.012±0.025(19)	0.217±0.017(19)^*^	0.205±0.019(19)^*^	2.49±0.25(19)^*^	517.60±26.78(19)^*^	5.51±0.34(19)^*^
WT (+PKA)	6.0±0.00(9)^*^^†^	0.463±0.013(9)^†^	−0.093±0.010(9) ^*^^†^	0.226±0.022(9)^*^	0.319±0.028(9) ^*^^†^	4.62±0.34(9) ^*^^†^	784.92±70.90(9)^†^	7.45±0.49(9) ^*^^†^
3SA (−PKA)	6.1±0.00(19)^*^	0.535±0.020(19)^*^	0.074±0.018(19)^¶^	0.185±0.08(19)^*^	0.111±0.011(19) ^*^^¶^	3.19±0.46(19)^*^	312.05±18.53(19)^¶^	6.18±0.37(19)^*^
3SA (+PKA)	5.98±0.02(8)^*^^†^	0.512±0.024(8)^*^	0.043±0.010(8) ^*^^€^	0.196±0.014(8)^*^	0.153±0.020(8) ^*^^€^	3.53±0.35(8)^*^	333.95±35.80(8)^*^^€^	4.95±0.61(8) ^*^^€^

*P1, XB stiffness; P2, minimum force attained at the end of Phase 2 of the stretch-activation response, an index of the magnitude of XB detachment; P3, the new steady-state force attained in response to the imposed stretch in muscle length; an index of force enhancement above initial pre-stretch isometric levels; P_df_, the trough-to-peak amplitude of the delayed force development phase (Phase 3), an index of the magnitude of overall post stretch XB recruitment; P_0_, pre-stretch isometric force; k_tr_, rate of force redevelopment; k_rel_, rate of XB detachment into the non-force bearing state; k_df_, rate of delayed XB recruitment into the force-bearing state in Phase 3. A minimum of 3 hearts per group were used, and the number of preparations for each group is presented in brackets. Values are expressed as mean ± S.E.M*.

† *Significantly different vs. the corresponding (−PKA) group*.

¶ *Significantly different compared to the corresponding (−PKA) WT group*.

€ *significantly different compared to the corresponding (+PKA) WT group*.

* *Significantly different compared to the corresponding group at short SL; asterisks indicate P < 0.05*.

A notable finding was that *k*_rel_ was significantly slower by ~66 and ~88% in the 3SA group when compared to the WT group at long and short SL, respectively, under basal conditions (Figure 4A, Table 2). The slowing of *k*_rel_ in the 3SA group was also evident following PKA treatment: *k*_rel_ was ~135 and ~67% slower when compared to the WT group at long and short SL, respectively (Table 2). Collectively, our data demonstrate that ablation of basal cMyBP-C phosphorylation by itself is sufficient to significantly slow *k*_rel_, and the inability to phosphorylate cMyBP-C blunts the PKA-mediated accelerations in XB cycling rates.

### Effect of cMyBP-C phospho-ablation on length-dependent changes in the magnitude of XB detachment (P2)

The amplitude of P2 was measured to estimate the magnitude of XB detachment following stretch (see Materials and Methods; Figure 1A). As reported earlier (Stelzer et al., 2006d), PKA treatment decreased P2 values (i.e., produced more negative P2 values) at both SL's in the WT group but not in the 3SA group—leading to a significant PKA-cMyBP-C phosphorylation interaction effect (Table 2; Figure 1). The significant reduction of the PKA-mediated increase in the magnitude of XB detachment in 3SA myocardium following stretch suggests that cMyBP-C phosphorylation likely removes an inhibitory brake on XB cycling that facilitates accelerated XB detachment from actin.

### Effect of cMyBP-C phospho-ablation on length-dependent changes in muscle fiber stiffness (P1)

**Figure 5:**
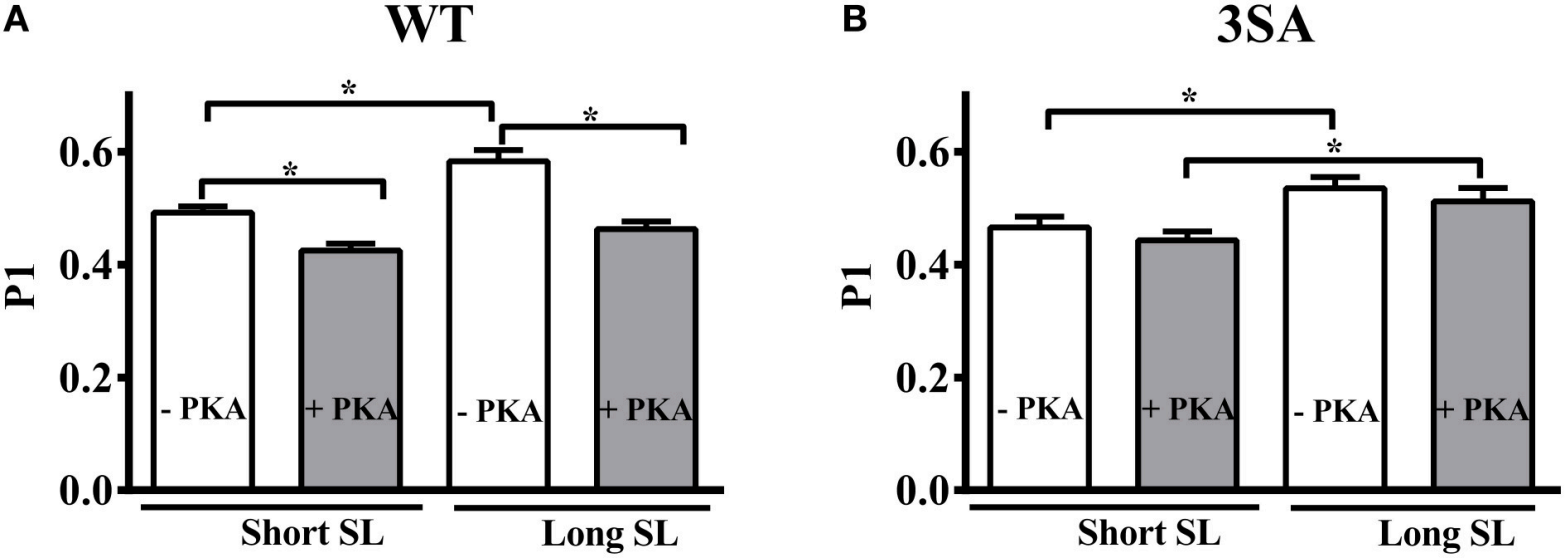
**Effect of cMyBP-C phospho-ablation on SL- and PKA-dependent changes in the magnitude of sudden-stretch induced increase in the XB stiffness (P1)**. P1 was calculated from the force responses elicited due to a sudden 2% stretch in muscle length imposed on isometrically-contracting myocardial preparations at ~35% of maximal Ca^2+^ activation level (Stelzer et al., 2006c) in **(A)** WT, and **(B)** 3SA myocardial preparations. PKA treatment significantly decreased P1 at both SL's in WT preparations but not in 3SA preparations—indicating that PKA-dependent changes in P1 are abolished in cMyBP-C phospho-ablated skinned myocardium. Values are expressed as mean ± S.E.M. The number of preparations used for each group are shown in Table 2. A minimum of 3 hearts per group were used with multiple preparations from each heart. ^*^P < 0.05.

We measured the magnitude of the elicited instantaneous increase in force in response to the imposed stretch (i.e., P1 in Figure 1), an index of muscle fiber stiffness (Mamidi et al., 2015). Under basal conditions, decreasing SL significantly decreased P1 by ~16% and ~13% in the WT and 3SA groups, respectively (Table 2). Most importantly, PKA treatment decreased P1 by ~21% and ~14% at long and short SL's in the WT, but had no effect on P1 in the 3SA group (Figure 5; Table 2)—contributing to the PKA-cMyBP-C phosphorylation interaction effect (Table 2).

### Effect of cMyBP-C phospho-ablation on length-dependent changes in the amplitude of phase 3

P3 is measured from pre-stretch steady state force to the peak value of the delayed force development in phase 3 (Figure 1A). P3 is the resultant new steady-state force attained in response to the imposed stretch and is due to the recruitment of additional XBs into the force-bearing state following the stretch in muscle length. Following PKA treatment, P_df_ values exceed P3 values (Stelzer et al., 2006c), as P2 values are often negative, therefore, under these conditions P_df_ represents the entire amplitude of Phase 3 taking into account all XBs recruited into the force-bearing state following acute stretch (Stelzer et al., 2006d). P3 was decreased by ~35% at short SL compared to long SL in WT group under both basal conditions and following PKA treatment (Table 2). Likewise, P3 decreased by ~31 and ~25% at short SL compared to long SL in 3SA group under basal conditions and following PKA treatment, respectively (Table 2). No significant differences were observed in P3 between WT and 3SA groups under any of the conditions studied.

P_df_ was increased by ~40 and ~60% at long SL compared to short SL in the WT group under basal conditions and following PKA treatment, respectively (**Figure 7**; Table 2). Likewise, P_df_ increased by ~61 and ~76% at long SL compared to short SL in the 3SA group under basal conditions and following PKA treatment, respectively (**Figure 7**; Table 2). As reported earlier (Stelzer et al., 2006d), P_df_ was significantly enhanced in WT skinned myocardium following PKA treatment at long and short SL (~56 and ~36%, respectively; Table 2), indicating that PKA phosphorylation increases the overall magnitude of XB recruitment when considering an increase in the number of detached XBs following stretch (i.e., increased P2). The amplitude of Phase 3 (P_df_) in the 3SA group was significantly lower compared to the WT group at both SL's under basal conditions, and following PKA treatment (**Figure 7**). The enhancement in P_df_ that was observed following PKA treatment at both SL's in the WT group, was not observed in the 3SA group (Table 2) resulting in a significant PKA-cMyBP-C phosphorylation interaction effect.

### Effect of cMyBP-C phospho-ablation on length-dependent changes in the rate of XB recruitment (*k*_df_)

**Figure 6:**
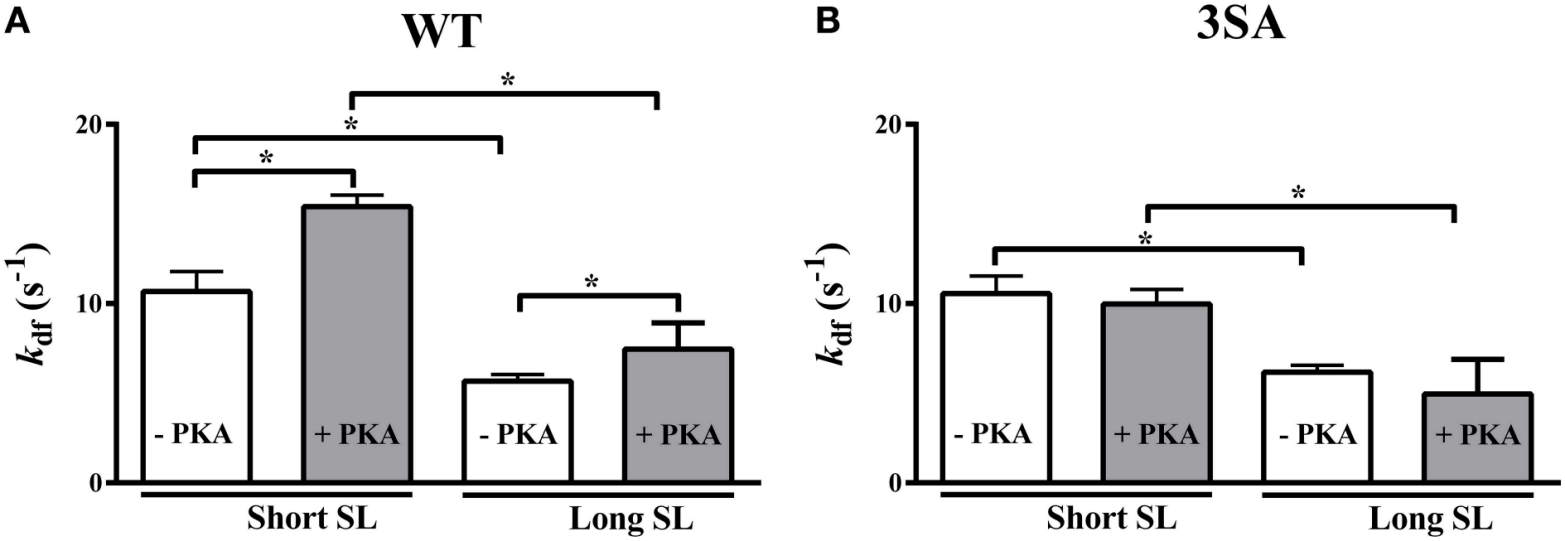
**Effect of cMyBP-C phospho-ablation on SL- and PKA-dependent changes in the rate of XB recruitment (***k***_**df**_)**. Isometrically-contracting myocardial preparations were subjected to a sudden 2% stretch in muscle length at ~35% of maximal Ca^2+^ activation level and the elicited force responses were used to measure *k*_df_ in **(A)** WT, and **(B)** 3SA myocardial preparations. Decreased SL accelerated *k*_df_ under basal conditions and following PKA treatment in both WT and 3SA preparations. PKA treatment significantly accelerated *k*_df_ at both short and long SL's in the WT group, but not in the 3SA group—indicating that PKA-induced accelerations in the rate of XB recruitment are abolished in cMyBP-C phospho-ablated skinned myocardium. Values are expressed as mean ± S.E.M. The number of preparations used for each group are shown in Table 2. A minimum of 3 hearts per group were used with multiple preparations from each heart. ^*^P < 0.05.

Decreasing SL significantly accelerated *k*_df_ by ~94 and ~71% in the WT and 3SA groups, respectively (Table 2; Figure 6). Following PKA treatment, *k*_df_ was significantly accelerated by ~107 and ~102% at short SL in the WT and 3SA groups, respectively (Table 2; Figure 6). Furthermore, PKA treatment significantly accelerated *k*_df_ by ~35% and by ~44% at long and short SL in the WT group, respectively (Figure 6A). Under basal conditions *k*_df_ was not different between the WT and 3SA groups at either SL (Table 2). In contrast to the WT group, PKA treatment did not induce acceleration in *k*_df_ in the 3SA group at either SL (Figure 6B). As a result, differences in *k*_df_ that were not apparent between the WT and 3SA groups under basal conditions became apparent following PKA treatment (Table 2)—resulting in a significant PKA-cMyBP-C phosphorylation interaction effect.

### Effect of cMyBP-C phospho-ablation on length-dependent changes in the rate of force redevelopment (*k*_tr_)

Decreasing SL significantly accelerated *k*_tr_ at submaximal Ca^2+^-activation in both the WT and 3SA groups under basal conditions, and following PKA treatment (Table 2). In addition, PKA treatment significantly accelerated *k*_tr_ at both SL's in the WT group, but a PKA-mediated acceleration in *k*_tr_ was not observed in the 3SA group (Table 2).

## Discussion

The heart is tuned to adjust its stroke volume to match systemic demands on a beat-to-beat basis. Increased sympathetic drive in response to increased circulatory demands enhances ventricular filling and results in increased cardiac output. Accordingly, the Frank-Starling relationship and its underlying mechanisms of LDA are influenced by an interplay between increased ventricular filling (corresponding to increased myofilament sarcomere length (SL)) and increased cardiac output, and are enhanced by increased β-adrenergic stimulation which leads to XB cycling (Tong et al., 2008). cMyBP-C is a principal target of increased β-adrenergic stimulation via PKA phosphorylation, however, the role of cMyBP-C phosphorylation in modulating length-dependent changes in XB kinetics in cardiac muscle is poorly understood. Impaired modulation of LDA is a common feature of human heart failure, and is often accompanied by reduced myofilament protein phosphorylation, including cMyBP-C (El-Armouche et al., 2007; Jacques et al., 2008; Copeland et al., 2010; Van Dijk et al., 2012). Because cMyBP-C phosphorylation has been shown to be cardioprotective (Sadayappan et al., 2006) and plays an important role in modulating XB kinetics (Stelzer et al., 2006d), cMyBP-C dephosphorylation could underlie the impaired LDA observed in conditions of heart failure. To determine the precise molecular mechanisms by which cMyBP-C phosphorylation regulates length-dependent changes in XB kinetics, we performed stretch-activation experiments in skinned myocardium isolated from WT and cMyBP-C phospho-ablated (i.e., 3SA) hearts, at variable SL, prior to and following PKA treatment. Results from this study provide the first evidence of a molecular mechanism by which decreased cMyBP-C phosphorylation results in impaired modulation of length-dependent changes in XB kinetics. Specifically, we demonstrate that cMyBP-C phospho-ablation significantly attenuated the acceleration of the rate of XB detachment due to reduced SL and PKA phosphorylation. Furthermore, cMyBP-C phospho-ablation significantly blunted the magnitude of cooperative XB recruitment and abolished PKA-mediated accelerations in the rate of XB recruitment (Table 2). These effects would be predicted to significantly reduce the ability of the heart to enhance its cardiac output in conditions of increased sympathetic drive, and suggest that decreased cMyBP-C phosphorylation contributes to a depressed Frank-Starling relationship, thereby contributing to impaired contractile function in failing hearts.

### Effect of cMyBP-C phospho-ablation on length-dependent changes in steady-state contractile function

It is well established that increased SL results in increased sensitivity of the myofilaments to Ca^2+^-activation (Kentish et al., 1986; Dobesh et al., 2002), and this phenomenon is modulated by PKA-mediated phosphorylation of myofilament contractile proteins (Konhilas et al., 2003; Wijnker et al., 2014). Furthermore, increased SL leads to increased force generation, in part by decreasing the distance between myosin heads and the thin filament, which enhances cooperative XB recruitment (Moss et al., 2004) because of an increased probability of actomyosin interaction due to the closer juxta-position of XB to the thin filament. In this context, we have recently shown that skinned myocardium lacking cMyBP-C, which results in a reduced distance between actin and myosin that promotes XB binding to the thin filament (Colson et al., 2007), displays a blunted reduction of the Ca^2+^-sensitivity of force generation due to decreases in SL (Mamidi et al., 2014). Using skinned myocardium lacking cMyBP-C, previous studies have suggested a role for cMyBP-C in modulating reductions in Ca^2+^-sensitivity of force due to decreased SL and PKA treatment (Cazorla et al., 2006; Chen et al., 2010). A recent study utilized myocytes expressing phospho-ablated cMyBP-C and demonstrated that cMyBP-C contributes to changes in myofilament Ca^2+^-sensitivity when SL is varied between 1.9 and 2.3 μm (Kumar et al., 2015). The molecular mechanisms for these effects are unclear, however, it was proposed that blunted length-dependent changes in pCa_50_ following cMyBP-C phospho-ablation may be related to a disruption in the binding of the N-domain of cMyBP-C to actin, for which it competes with both the inhibitory domain of cTnI and myosin heads in a phosphorylation and length-dependent manner (Kumar et al., 2015). We found no significant differences in the maximal force generation, half-maximal force (i.e., pCa_50_) and cooperativity of force generation between 3SA and WT skinned myocardium at baseline (no PKA) or following PKA treatment (Table 1) within a range of SL of 1.9–2.1 μm. Furthermore, in contrast to earlier studies (Cazorla et al., 2006; Lee et al., 2010), we found that length-dependent increases in Ca^2+^-sensitivity when SL was increased were abolished following PKA treatment in both WT and 3SA skinned myocardium (Table 1). The reasons for these disparities are unclear; however, they may be due to the narrower range of SL employed in the present study (1.9–2.1 μm) compared to previous studies (1.9–2.3 μm), which may have reduced the overall magnitude of the shift in Ca^2+^-sensitivity when SL is increased. However, in agreement with a recent study (Kumar et al., 2015), we observed a main effect of cMyBP-C phosphorylation on length-dependent changes in Ca^2+^-sensitivity. This suggests that abolishing cMyBP-C phosphorylation blunts the reduction of the myofilament sensitivity to Ca^2+^ (pCa_50_) in response to reduced SL (Table 1).

### cMyBP-C phospho-ablation significantly slows the basal rate of XB detachment (*k*_rel_) and abolishes the SL- and PKA-based effects on *k*_rel_

Because *k*_rel_ is the rate limiting step in the XB cycle assuming a two-state XB model and under loaded conditions (Strang et al., 1994), *k*_rel_ is a key determinant of *in vivo* force generation (Biesiadecki et al., 2014). Changes in the rate of XB detachment affect XB cycling at the myofilament level, and consequently impact rates of systolic pressure development and diastolic pressure relaxation at the whole-heart level (Gresham et al., 2014; Gresham and Stelzer, 2016). Previous studies have shown that *k*_rel_ is significantly accelerated following PKA-mediated cMyBP-C phosphorylation (Stelzer et al., 2006d), and that cMyBP-C phospho-ablation eliminates the PKA-mediated acceleration of the rate of XB detachment (Tong et al., 2008). In addition, we recently showed that ablation of cMyBP-C prevents the acceleration in *k*_rel_ due to decreased SL that is observed in WT skinned myocardium (Mamidi et al., 2014), suggesting that cMyBP-C is required for length-dependent modulation of XB detachment from the thin filament. Measurements from human heart failure samples suggest that cMyBP-C phosphorylation can also contribute to length-dependent acceleration of the rate of XB detachment, as myocardium isolated from patients with hypertrophic cardiomyopathy (HCM) displayed reduced cMyBP-C phosphorylation exhibited impaired LDA (Sequeira et al., 2013). However, the mechanism by which cMyBP-C phosphorylation modulates the length-dependent acceleration of the rate of XB detachment in the myocardium remains unclear.

In this study, we show that cMyBP-C phospho-ablation significantly slows *k*_rel_ at long and short SL, both at baseline and following PKA treatment (Table 2) when compared to WT skinned myocardium (Figure 4). As shown previously (Tong et al., 2008), we also observed a PKA-mediated acceleration of *k*_rel_ at long SL in WT skinned myocardium that was abolished in 3SA skinned myocardium (Table 2). PKA treatment reduced XB stiffness (P1) (Figure 5) and produced more negative values for P2 in WT skinned myocardium indicating that PKA phosphorylation facilitates XB detachment from the thin filament. It has been suggested that P2 amplitude is an indicator of the reversal of the force-generating step following stretch, such that more negative P2 values are indicative of enhanced reversal of the phosphate release step during muscle contraction (Davis and Epstein, 2003). Enhanced reversibility of force producing steps in the XB cycle may result in a functional advantage in the myocardium because XBs can quickly detach and reattach to the thin filament without increasing ATP utilization and energy consumption (Davis and Epstein, 2003). Thus, the lack of PKA-mediated decreases in P2 in 3SA skinned myocardium may increase energy utilization and decrease contractile efficiency, contributing to development of hypertrophy and systolic dysfunction *in vivo*.

Slowed XB detachment due to cMyBP-C phospho-ablation would increase the overall dwell time (i.e., duty ratio) of XBs bound to the thin filament and consequently delay thin filament deactivation, which can alter the timing of ventricular relaxation (Hanft et al., 2008; Biesiadecki et al., 2014). Delayed thin filament deactivation would be predicted to prolong diastolic relaxation and slow ventricular filling *in vivo* (i.e., cause diastolic dysfunction) during basal function and following increased β-adrenergic stimulation. Indeed, we recently showed that 3SA mouse hearts displayed a prolonged time course of pressure relaxation and an impaired enhancement of the rate of pressure relaxation in response to β-adrenergic stimulation (Gresham and Stelzer, 2016). Similarly, a recent study (Rosas et al., 2015) demonstrated that impaired ventricular relaxation in 3SA hearts is not due alterations in intracellular Ca^2+^ transients, strongly supporting our hypothesis that slowed XB detachment is the predominant factor underlying diastolic dysfunction in 3SA mice (Gresham and Stelzer, 2016). Collectively, our data implicates cMyBP-C phosphorylation as a physiological modulator of lusitropy in the heart (Lewinter and Palmer, 2015).

### cMyBP-C phospho-ablation blunts PKA-mediated accelerations in the rate of XB recruitment (*k*_df_) and magnitude of the delayed force transient (P_df_)

**Figure 7:**
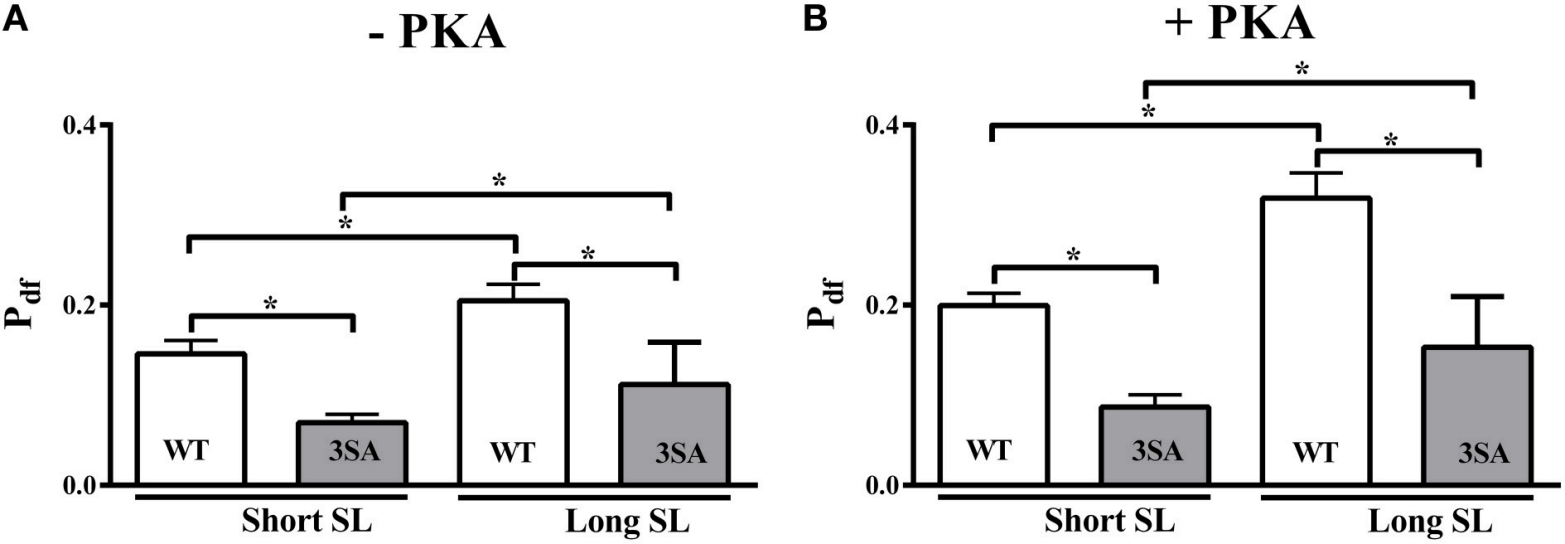
**Effect of cMyBP-C phospho-ablation on SL- and PKA-dependent changes in the magnitude of stretch-induced XB recruitment (P_**df**_)**. Isometrically-contracting myocardial preparations were subjected to a sudden 2% stretch in their muscle length at ~35% of maximal Ca^2+^ activation level and the elicited force responses were used to measure **(A)** P_df_ under basal conditions (-PKA) and **(B)** P_df_ following PKA treatment at short and long SL's in WT (white bars) and 3SA (gray bars) groups. P_df_ was significantly lower at short SL compared to long SL in the WT and 3SA groups under basal conditions and following PKA treatment. However, P_df_ was significantly lower in the 3SA group compared to WT group at both SL's under basal conditions and following PKA treatment—indicating that the overall number of XBs being recruited into the force-bearing state in response to a stretch in muscle length is significantly decreased in cMyBP-C phospho-ablated skinned myocardium. Values are expressed as mean ± S.E.M. The number of preparations used for each group are shown in Table 2. A minimum of 3 hearts per group were used with multiple preparations from each heart. ^*^P < 0.05.

It is known that PKA phosphorylation of cMyBP-C enhances the rate (*k*_rel_) and magnitude (P2) of XB detachment (Stelzer et al., 2006d), thereby increasing the number of available actin binding sites for recruitment and binding of detached or non-cycling XBs following stretch. Therefore, the absence of PKA-mediated accelerations in *k*_rel_ or enhancement in P2 at short SL in 3SA skinned myocardium (Figure 4A, Table 2) would be predicted to also reduce the overall magnitude of XB recruitment during Phase 3 (i.e., P_df_), and consequently impair systolic pressure development and ejection *in vivo* following β-adrenergic stimulation (Gresham and Stelzer, 2016). Additionally, impairments in systolic function can directly arise from defects in XB recruitment mechanisms at the myofilament level. Consistent with a recent study (Mamidi et al., 2014), here we observed that the rate of XB recruitment (*k*_df_) was significantly accelerated at short SL in both WT and 3SA skinned myocardium (Table 2, Figure 7A). PKA-mediated phosphorylation of cMyBP-C significantly accelerated *k*_df_ at long and short SL in WT skinned myocardium (Figure 7A), however, PKA-induced accelerations in *k*_df_ were abolished in 3SA skinned myocardium (Figure 7B), such that following PKA phosphorylation, *k*_df_ in 3SA skinned myocardium was significantly slower compared to WT skinned myocardium (Table 2). To determine if cMyBP-C phosphorylation also affects the number of XBs recruited into the force-bearing state in response to changes in SL, we measured the amplitude of the Phase 3 delayed force redevelopment (Stelzer and Moss, 2006; Figure 2A). The delayed force redevelopment in Phase 3 following stretch activation is considered to be primarily due to the recruitment of additional XBs into the force-bearing states (Campbell et al., 2004; Linari et al., 2004; Stelzer et al., 2006c), and varies with the level of Ca^2+^-activation (Stelzer et al., 2006c). In the present study we found no differences in P3 between WT and 3SA groups indicating that the number of additional XBs recruited above initial pre-stretch steady-state force was similar (Figure 1A), when the activation levels were matched. However, because PKA treatment often results in more negative values in P2 (i.e., greater magnitude of XB detachment, Stelzer et al., 2006d), the entire magnitude of XB recruitment of Phase 3 following acute stretch is represented by P_df_ which takes into account the point of transition in the stretch activation transient where XB recruitment begins to dominate (i.e., P2, Stelzer et al., 2007a; Figure 1A). Our data show that P_df_ was significantly reduced in 3SA skinned myocardium under all conditions tested when compared to WT skinned myocardium (Figure 6; Table 2), indicating that cMyBP-C phospho-ablation significantly blunts (Table 2) the overall magnitude of stretch-induced XB recruitment at submaximal Ca^2+^ activations.

A blunting in the rate and magnitude of XB recruitment due to cMyBP-C phospho-ablation may be due to the fact that cMyBP-C phosphorylation alters the dynamic binding between the thick and thin filaments. cMyBP-C phosphorylation likely relieves a constraint on myosin heads (Colson et al., 2012; Pfuhl and Gautel, 2012) which enhances the probability of actomyosin interactions and accelerates the spread of cooperative XB recruitment and activation throughout the thin filament (Kampourakis et al., 2014; Moss et al., 2015) to accelerate the rate of force development (Moss et al., 2015). Therefore, depressed rates and magnitude of XB recruitment following PKA treatment in cMyBP-C phospho-ablated skinned myocardium would be predicted to significantly slow the rate of force generation at the myofilament level and diminish the rate of systolic pressure generation. Indeed, hemodynamic measurements of pressure-volume relationships in 3SA mice showed a significant slowing in achieving peak pressure development, and a blunted acceleration in the rate of pressure development (i.e., reduced dp/dt_max_) following infusion of the β-agonist dobutamine compared to WT mice (Gresham and Stelzer, 2016). Furthermore, radio-telemetry measurements of *in vivo* pressure generation in unanesthetized 3SA mice revealed a significant decrease in the magnitude of peak pressure generation compared to WT mice in response to an acute dobutamine challenge (Gresham and Stelzer, 2016). Collectively, these findings from *in vivo* studies in 3SA hearts correlate well with a decreased magnitude of XB recruitment (i.e., decreased P_df_) in 3SA myocardium at the molecular level (Figure 7; Table 2). The diminished rate and magnitude of XB recruitment in 3SA skinned myocardium observed here (Figures 6, 7) in conjunction with reduced rates and magnitude of peak pressure development following dobutamine infusion *in vivo* (Gresham and Stelzer, 2016), suggests that cMyBP-C phospho-ablation decreases LV peak power generation and cardiac output. Peak power output in cardiac muscle depends on the number of force-generating XBs (Mcdonald, 2011), and is enhanced when strongly-bound XBs prolong thin filament activation thereby facilitating the recruitment of adjacent XBs into force-generating states by cooperative mechanisms (Hinken and Solaro, 2007; Mcdonald, 2011). Thus, cMyBP-C phospho-ablation appears to decrease peak power output by slowing the rate of cooperative XB recruitment and limits the number of XBs recruited to the thin filament.

## Conclusions

Results from the present study demonstrate that cMyBP-C phosphorylation modulates LDA because cMyBP-C phospho-ablation resulted in a blunting of SL-dependent decreases in myofilament Ca^2+^-sensitivity of force generation (pCa_50_), and accelerations in the rate of XB detachment (*k*_rel_) in 3SA skinned myocardium. These findings suggest that along with cTnI phosphorylation, cMyBP-C phosphorylation also contributes to the modulation of myofilament steady-state force (Chen et al., 2010; Kumar et al., 2015). Furthermore, our data show that PKA phosphorylation of both cMyBP-C and cTnI in WT skinned myocardium result in significant accelerations of the rates of XB detachment (*k*_rel_) and XB recruitment (*k*_df_), and enhancements in the magnitudes of XB detachment (P2) and recruitment (P_df_). In contrast, in 3SA cMyBP-C phospho-ablated skinned myocardium where PKA phosphorylation can only phosphorylate cTnI, we observed a significant blunting in PKA-mediated accelerations of the rates of XB detachment (*k*_rel_) and XB recruitment (*k*_df_), and enhancements in the magnitudes of XB detachment (P2) and recruitment (P_df_). Thus, cMyBP-C phosphorylation appears to be the predominant modulator of the rates of XB detachment and recruitment. Low angle X-ray diffraction studies reveal that XBs are displaced toward the actin filament to a similar extent in WT skinned myocardium where both cTnI and cMyBP-C are phosphorylated by PKA, as in cTnI PKA-phospho-ablated skinned myocardium in which only cMyBP-C can be phosphorylated (Colson et al., 2012). This suggests that the radial disposition of XBs is primarily mediated by cMyBP-C phosphorylation. The closer juxtaposition of XBs to actin due to cMyBP-C phosphorylation has been proposed to be due to a release of the constraint imposed by cMyBP-C on myosin heads which increases the probability of XB binding to actin and accelerates cooperative XB recruitment (*k*_df_) and binding to actin (Colson et al., 2008). The lack of acceleration of *k*_df_ following PKA phosphorylation in 3SA skinned myocardium along with a diminished magnitude of XB recruitment (P_df_) would be expected to impair force generation in conditions of increased β-adrenergic stimulation *in vivo* (Gresham and Stelzer, 2016). Furthermore, cMyBP-C phospho-ablation prevents the acceleration in the rates and magnitude of XB detachment (*k*_rel_ and P2, respectively) following PKA phosphorylation and decreased SL (when the cardiac muscle is shortening during systolic ejection), which would be expected to delay the force decay in late-systole, thereby, slowing cardiac pressure relaxation (Gresham and Stelzer, 2016). The net effect of the acceleration of XB detachment and recruitment due to cMyBP-C phosphorylation is to decrease XB duty ratio, which along with cTnI phosphorylation, contributes to enhanced thin filament deactivation and diminished force generation.

Our data demonstrate that cMyBP-C phosphorylation regulates length-dependent XB kinetics, by modulating the rate and magnitude of XB detachment and recruitment. Indeed, we show that cMyBP-C phospho-ablation prevents SL- and PKA-induced enhancements in the rate and magnitude in XB detachment, and PKA-induced enhancements in the rate and magnitude of XB recruitment. Failing human hearts have a decreased capacity for increasing cardiac output in conditions of increased systemic demand (Holubarsch et al., 1996), partly because of a depressed Frank-Starling relationship. The molecular basis for impaired Frank-Starling relationships has been proposed to be due to decreased PKA-mediated myofilament protein phosphorylation (Van Der Velden et al., 2003; Hanft and Mcdonald, 2009). Here we provide explicit evidence which shows that cMyBP-C phosphorylation is an important regulator of length-dependent changes in XB kinetics and that decreased cMyBP-C phosphorylation may be an underlying mechanism for depressed Frank-Starling relationships and cardiac output in human HF. Recent studies have shown that in human hypertension, which is a common precursor for development of heart failure with preserved ejection fraction (HFpEF), cMyBP-C phosphorylation is reduced and may contribute to a prolongation of XB attachment time at the myofilament level, thereby impairing diastolic relaxation (Donaldson et al., 2012). Our finding that cMyBP-C phospho-ablation significantly slows the rate of XB detachment provides evidence that indeed, decreased cMyBP-C phosphorylation in hypertension may be partially responsible for slowed myofilament XB relaxation and LV diastolic function. Therefore, our data show that manipulation of cMyBP-C phosphorylation levels may be an attractive therapeutic strategy for modulating systolic and diastolic contractile dysfunction in HF.

## Author contributions

All the experiments were done at the Department of Physiology and Biophysics, Case Western Reserve University, Cleveland, Ohio, USA. RM, KG, and JS participated in performing the experiments and data collection, the conception and design of the experiments, the analysis and interpretation of data, and in writing and revising the manuscript. SV participated in data analysis, interpretation of data, writing, and revising the manuscript. All authors approved the final version of this manuscript.

### Conflict of interest statement

The authors declare that the research was conducted in the absence of any commercial or financial relationships that could be construed as a potential conflict of interest.

## Abbreviations

cMyBP-C: cardiac myosin binding protein-C
WT: wild-type
XB: cross-bridge
*k*_tr_: rate of force redevelopment
*k*_rel_: rate of XB detachment; P2: magnitude of XB detachment
*k*_df_: rate of XB recruitment
P_df_: magnitude of delayed force development
PKA: protein kinase A.
